# Corrigendum: Building a realistic, scalable memory model with independent engrams using a homeostatic mechanism

**DOI:** 10.3389/fninf.2024.1461597

**Published:** 2024-08-09

**Authors:** Marvin Kaster, Fabian Czappa, Markus Butz-Ostendorf, Felix Wolf

**Affiliations:** ^1^Laboratory for Parallel Programming, Department of Computer Science, Technical University of Darmstadt, Darmstadt, Germany; ^2^Data Science, Translational Medicine and Clinical Pharmacology, Boehringer Ingelheim Pharma GmbH & Co. KG, Biberach, Germany

**Keywords:** learning, memory, homeostatic plasticity, structural plasticity, scalable

In the published article, there was an error in Figure 7 as published. The wrong image was included. Figures 7 and 8 were identical! The corrected Figure 7 and its caption appear below.

**Figure 7:**
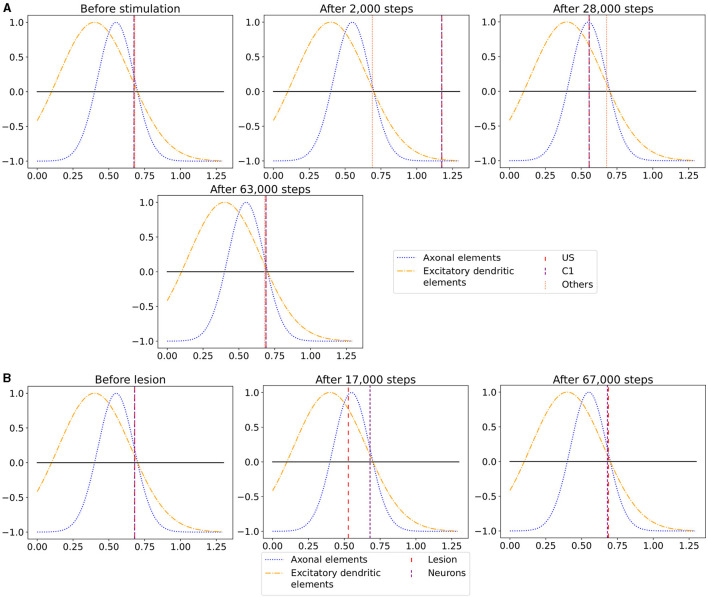
The average calcium level of groups of neurons of a single box (*x*-axis) with calcium-dependent growth curves (*y*-axis). **(A)** Calcium level before and after the ensembles US and C1 were stimulated at step 450,000. Subsequentially, their calcium levels increased and growth curves caused synaptic elements to decrease and synapses to prune. When stimulation stopped and synapses were pruned, activities and calcium levels, respectively, dropped below the homeostatic set-point which, in turn, triggered the growth of synaptic elements and potentially also the growth of new synapses. Note, that calcium levels below the set-point were in an optimal regime for axonal element formation while dendritic elements grew slower. A surplus of axonal elements may result in more long-range connections while a prolongued growth of dendritic elements extended the phase in which new engrams could form because it may take longer until activities return to a homeostatic set-point. **(B)** Average calcium levels during ablation studies in which we removed connectivity of 50% of the neurons in a box. Directly after the stimulation, calcium levels of lesioned neurons dropped due to the lack of input. As a result, the neurons start regrowing synaptic elements until enough synapses were formed to restore activity homeostasis. The homeostatic reorganization is comparable to engram formation after stimulation in A. Note, that even for higher deletion rates neurons will return average firing rates to the homeostatic set-point (data not shown) very much as in B, however without functional recovery of trained engrams.

In the published article, there was an error in Supplementary Figures S1 and S2. The order of the figures was reversed. The corrected order of the Figures S1 and S2 have been updated in the original article. References to the supplementary material in the main text remain unchanged.

In the published article, there was an error. There was a missing minus sign in Equation 5.

A correction has been made to **2 Materials and methods**, ***2.1 Model of structural*** ***plasticity***, *2.1.2 Forming and pruning of synapses*, **Equation 5**. This equation previously stated:


(5) $$\begin{array}{l} k_{i,j}=\exp\left(\frac{||x_{j}-x_{i}{||}_{2}^{2}}{\sigma^{2}}\right) \end{array}$$

The corrected equation appears below:


(5) $$\begin{array}{l} k_{i,j}=\exp\left(-\frac{||x_{j}-x_{i}{||}_{2}^{2}}{\sigma^{2}}\right) \end{array}$$

The authors apologize for this error and state that this does not change the scientific conclusions of the article in any way. The original article has been updated.

