# Supplementary material for: Building a realistic, scalable memory model with independent engrams using a homeostatic mechanism
Source: Front Neuroinform. 2024 Apr 19;18:1323203. doi: 10.3389/fninf.2024.1323203 (PMC11066267; doi:10.3389/fninf.2024.1323203)
Supplement: Supplementary file 1 [file Data_Sheet_1.pdf]

Supplementary Material

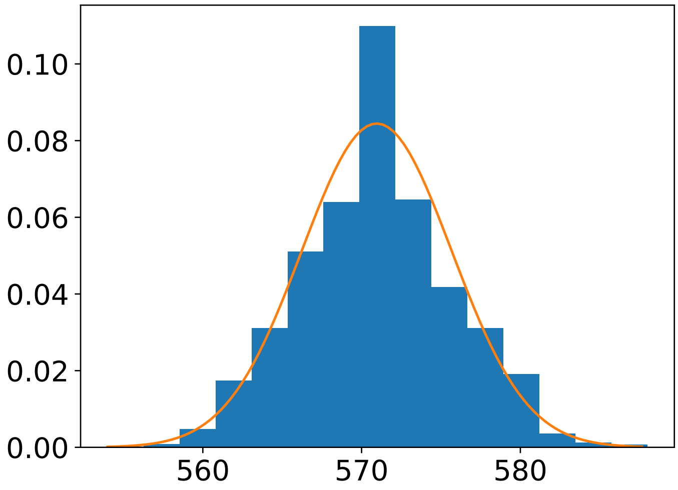

Figure S1. Histogram of the recorded firing rates of the readout neurons (blue bars) and the assumed normal distribution (orange lines) with a mean of $570.95\,\mathrm{Hz}$ and a standard deviation of 4.72.

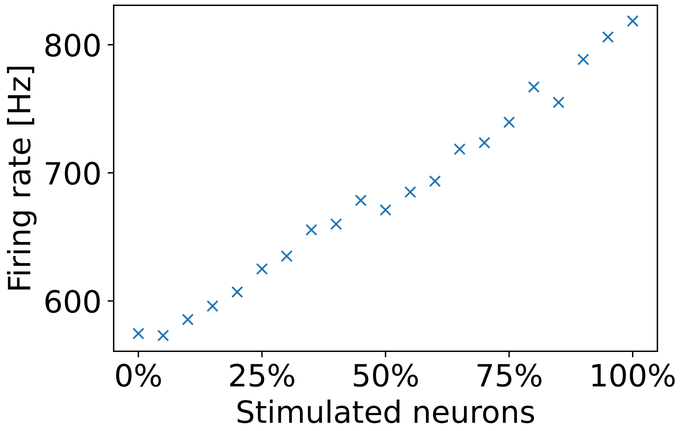

Figure S2. Firing rate during pattern retrieval. The normal firing rate of the readout neuron without any stimulation has a mean of $\mu = 574.5\,\mathrm{Hz}$ and a standard deviation $\sigma = 29.3$. When we stimulated an increasing number of neurons in the ensemble C1, the firing rate of the readout neuron of the associated readout neuron fired at an increasing rate. There are three exception were an increase of the number of stimulated neurons about 5% do not increase the firing rate.
